# The Diverse AAA+ Machines that Repair Inhibited Rubisco Active Sites

**DOI:** 10.3389/fmolb.2017.00031

**Published:** 2017-05-19

**Authors:** Oliver Mueller-Cajar

**Affiliations:** School of Biological Sciences, Nanyang Technological UniversitySingapore, Singapore

**Keywords:** Rubisco, activase, photosynthesis, AAA+ proteins, molecular chaperones, carbon fixation

## Abstract

Gaseous carbon dioxide enters the biosphere almost exclusively via the active site of the enzyme ribulose 1,5-bisphosphate carboxylase/oxygenase (Rubisco). This highly conserved catalyst has an almost universal propensity to non-productively interact with its substrate ribulose 1,5-bisphosphate, leading to the formation of dead-end inhibited complexes. In diverse autotrophic organisms this tendency has been counteracted by the recruitment of dedicated AAA+ (ATPases associated with various cellular activities) proteins that all use the energy of ATP hydrolysis to remodel inhibited Rubisco active sites leading to release of the inhibitor. Three evolutionarily distinct classes of these Rubisco activases (Rcas) have been discovered so far. Green and red-type Rca are mostly found in photosynthetic eukaryotes of the green and red plastid lineage respectively, whereas CbbQO is associated with chemoautotrophic bacteria. Ongoing mechanistic studies are elucidating how the various motors are utilizing both similar and contrasting strategies to ultimately perform their common function of cracking the inhibited Rubisco active site. The best studied mechanism utilized by red-type Rca appears to involve transient threading of the Rubisco large subunit C-terminal peptide, reminiscent of the action performed by Clp proteases. As well as providing a fascinating example of convergent molecular evolution, Rca proteins can be considered promising crop-improvement targets. Approaches aiming to replace Rubisco in plants with improved enzymes will need to ensure the presence of a compatible Rca protein. The thermolability of the Rca protein found in crop plants provides an opportunity to fortify photosynthesis against high temperature stress. Photosynthesis also appears to be limited by Rca when light conditions are fluctuating. Synthetic biology strategies aiming to enhance the autotrophic CO_2_ fixation machinery will need to take into consideration the requirement for Rubisco activases as well as their properties.

## The curious case of rubisco

The vast majority of carbon dioxide entering the living world does so via the slow and non-specific enzyme ribulose 1,5-bisphosphate carboxylase/oxygenase (Rubisco) (Spreitzer and Salvucci, 2002). The realization that this enzyme often represents the rate-limiting step of photosynthesis has made it a long-standing target for crop improvement strategies (Parry et al., 2007; Whitney et al., 2011a; Ort et al., 2015; Sharwood et al., 2016b). The peculiar properties of Rubisco can be understood as an accident of natural history. A highly complex reaction mechanism for ribulose 1,5-bisphosphate (RuBP) carboxylation evolved once in a high CO_2_ atmosphere lacking O_2_ (Andrews and Lorimer, 1987, Figure 1A). The unprecedented increase in atmospheric oxygen following the evolution of oxygenic photosynthesis increased the propensity of RuBP oxygenation, making it physiologically relevant (Andrews et al., 1973; Tcherkez, 2016). This resulted in massive metabolite damage (Linster et al., 2013) in the form of a build-up of 2-phosphoglycolate, which in contemporary plants is repaired by photorespiration (Bauwe et al., 2010). In C3 plants exposed to the current atmospheric environment, photorespiration operates at ~20% of photosynthesis (Cegelski and Schaefer, 2006), making it the second highest flux pathway. Operation of the photorespiratory pathway is energetically wasteful, resulting in a high selection pressure to reduce its flux. However, Rubisco's extensive adaptive walks through sequence space were not rewarded by the discovery of catalytic solutions that eliminated oxygenation (Maynard Smith, 1970; Mueller-Cajar and Whitney, 2008a). Instead it appeared easier to evolve a myriad of diverse syndromes that concentrate CO_2_ at the active site of the carboxylase (Badger et al., 1998; Rae et al., 2013; Sage, 2013). However, all of these mechanisms involve active transport, and thus increase the metabolic cost per CO_2_ fixed. Therefore, there was a concomitant pressure to enhance the catalytic fidelity of the enzyme by increasing its CO_2_/O_2_ specificity, as manifested most strongly in C3 plants and red algae (Tcherkez et al., 2006).

**Figure 1:**
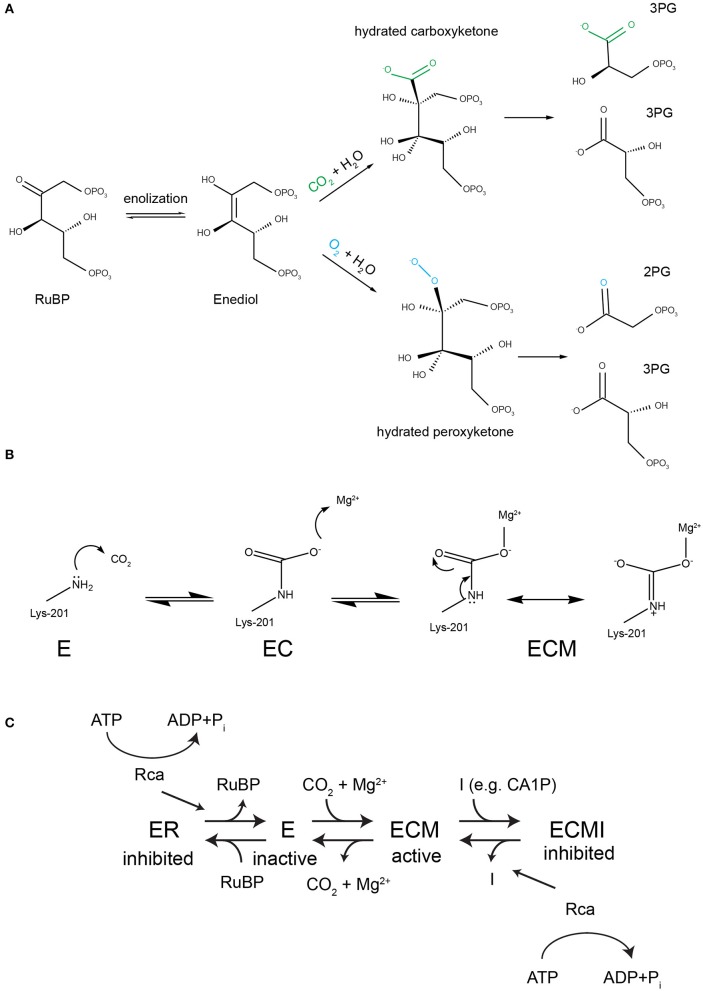
**Rubisco's reaction mechanism and its inhibition properties. (A)** A complex conserved reaction mechanism evolved to carboxylate ribulose 1,5-bisphosphate. The enediol intermediate can react with both oxygen and carbon dioxide. If oxygenation occurs the toxic metabolite 2-phosphoglycolate (2PG) is generated, which must be subjected to metabolite repair. **(B)** To perform the carboxylase reaction a conserved active site lysine (Lys-201 in spinach RbcL) must react with a non-substrate CO_2_ to form a carbamate (EC), followed by the binding of a Mg^2+^ ion to form the catalytically competent holoenzyme ECM. **(C)** Both the inactive apo (E) and the active holoenzyme (ECM) are prone to dead-end inhibition by sugar phosphates such as RuBP, which binds to E and CA1P (2-carboxy-D-arabinitol 1-phosphate), which binds to ECM. Rubisco activases (Rca) recognize inhibited active sites and use the energy of ATP hydrolysis to cause a conformational change that releases the inhibitor.

## Evolution of higher catalytic fidelity by rigidification of the active site

Catalysis by all Rubiscos requires two cofactors to bind at the active site permitting the functional holoenzyme to form (Figure 1B). A non-substrate CO_2_ reacts with the amine group of the conserved Lys-201 residue (spinach RbcL numbering) to form a carbamate. A Mg^2+^ ion is then bound to complete the activation process, forming the holoenzyme termed ECM (Lorimer et al., 1976; Cleland et al., 1998). The activated enzyme then binds the substrate RuBP, which is processed via a series of five partial reactions to eventually yield two molecules of 3-phosphoglycerate (3-PG) if carboxylated (Tcherkez, 2013, Figure 1A). The similarity in size and electrostatic potential of the gases CO_2_ and O_2_ (Kannappan and Gready, 2008) has culminated in a situation where the enzyme is unable to perfectly discriminate between the carboxylation substrate CO_2_ and the competing O_2_. The critical step at which the enzyme can influence the partitioning between carboxylation and oxygenation is during attack of the gaseous substrate by the enolized RuBP (Chen and Spreitzer, 1992). An analysis of decades of kinetic and isotope-fractionation data suggested that this task is achieved by a relative stabilization of the transition state for CO_2_, compared to O_2_ addition (Tcherkez et al., 2006). This stabilization manifests itself in both reduced flexibility of the active site and tighter binding of the carboxylated product (Pearce and Andrews, 2003). A well-documented outcome of this strategy is the trade-off where faster enzymes tend to exhibit higher Michaelis constants for CO_2_ and are less able to discriminate between CO_2_ and O_2_ (Bainbridge et al., 1995; Tcherkez et al., 2006; Savir et al., 2010). However, it is important to note that new Rubisco kinetic data is highlighting exceptions to these rules, at least regarding some algal enzymes exhibiting relatively low carboxylase efficiencies (Young et al., 2016).

## The emerging requirement for catalytic chaperones

A consequence of the described strategy, which tends to be less well popularized, relates to the tendency of the enzyme to become irreversibly inhibited by sugar phosphates. Since the unactivated apo-enzyme (E) already possesses all of the features required to bind the substrate RuBP, the active site will close when it encounters the substrate (Jordan et al., 1983; Duff et al., 2000). In the absence of the co-factors required to catalyze carboxylation or oxygenation, RuBP cannot be processed and is now bound unproductively, or “caught in the Rubisco mousetrap” (Andrews, 1996), to form Enzyme-RuBP (ER) (Figure 1C). At the same time, losing a valuable active site has reduced the capacity for carbon fixation of the host organism. RuBP is not the only inhibitory substrate, a palette of other sugar phosphates, including some generated by misfire-reactions of Rubisco itself, also tightly bind to the active site (Parry et al., 2008; Andralojc et al., 2012; Bracher et al., 2015). The affinity of the inhibitors is correlated with the enzyme's catalytic parameters, and based on the data available “superior” high specificity Rubiscos bind RuBP and other sugar phosphates more tightly than the low specificity enzymes with more flexible active sites (Pearce and Andrews, 2003; Pearce, 2006).

Over time, as Rubisco active sites became more and more adept at tightly binding the carboxylation-intermediate, the propensity for the apo-Enzyme to bind the substrate non-productively also increased (Pearce and Andrews, 2003). This led to a temporary removal of significant proportions of active sites from the pool of the enzyme. This problem could be alleviated by the action of molecular chaperones that would selectively engage inhibited Rubisco, and by performing a “chiropractic” maneuver (Carmo-Silva and Salvucci, 2011) conformationally reset the active site.

Earlier articles have comprehensively reviewed our knowledge on biochemical and physiological aspects of both the green-type (Portis, 1995, 2003; Portis et al., 2008; Carmo-Silva et al., 2015) and the red-type activase (Mueller-Cajar et al., 2014; Hauser et al., 2015b). Here we aim to direct attention toward the recent realization that in different autotrophic lineages multiple activase classes have converged on the same biochemical function. We attempt to integrate our understanding regarding mechanistic similarities and differences toward a framework regarding the chaperone-mediated rearrangement of the highly conserved inhibited Rubisco active site.

## The evolution of rubisco and the three Rca classes

In spite of the single phylogenetic origin and highly conserved reaction chemistry of Rubisco, a number of highly distinct clades of Rubisco can be observed today (Tabita et al., 2008). All Rubiscos are comprised of ~55 kDa large subunits that assemble as anti-parallel dimers. Each dimer harbors two active sites formed by the β-barrel C-terminal domain of one subunit and the N-terminal domain (containing a 5-stranded mixed beta sheet) of the other (Knight et al., 1990). This basic functional unit is then often found to be assembled into higher oligomeric states.

**Figure 2:**
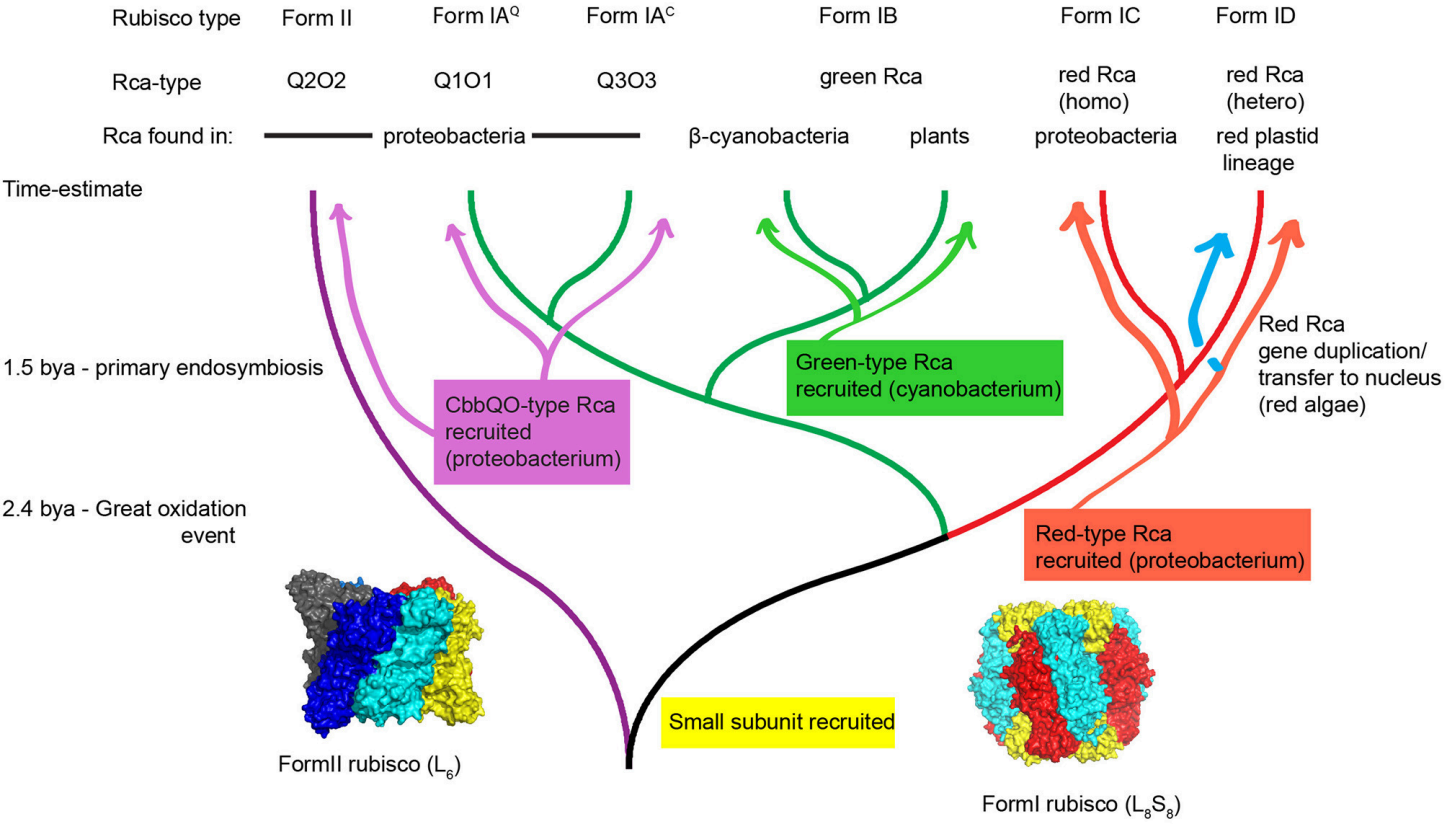
**Hypothetical scheme for the evolution of Rubisco and its activases**. Following the great oxidation event at least three different classes of Rubisco activase were recruited from the general molecular chaperone machinery toward a specialized Rubisco activase function in diverse autotrophic organisms. Green type and red-type Rca was maintained in eukaryotic phototrophs of the green and the red plastid lineage respectively. A phylogenetic tree was drawn using Rubisco large subunit sequences that are associated with activases. It is important to note that regarding non-red prokaryotic Rubisco sequences, many instances exist that do not have identifiable activase genes encoded in the same genome. Surface representations of a hexameric Form II Rubisco (pdb:4lf1) and spinach Form I Rubisco (pdb:8ruc) are shown. Structures shown in this paper were drawn using pymol.

Figure 2 shows a phylogenetic tree of selected RbcL sequences relevant to the present discussion about Rca. The last common ancestor of all extant Rubiscos was probably the aforementioned dimer of large subunits, and this arrangement is still found in a subset of the so-called Form II enzymes, such as the well-studied enzyme from *Rhodospirillum rubrum* (Anderson and Fuller, 1969). Contemporary Form II enzymes are often found to occupy higher order oligomeric states with a hexameric arrangement recently found to be common (Satagopan et al., 2014; Tsai et al., 2015; Varaljay et al., 2016). A key early innovation in Rubisco evolution concerned the recruitment of the small subunit, a ~15 kDa scaffolding protein that stabilized tetramers of dimers resulting in a L_8_S_8_ stoichiometry. These enzymes constitute the Form I clade of Rubiscos (Spreitzer, 2003). This clade branched early into a red (Form IC and D) and green-type branch (Form IA and B), the large subunits of which today maintain about 50% sequence identity to each other. Form IA Rubiscos can be subdivided into Form IA^Q^ and Form IA^C^ sequences, the latter always being associated with carboxysomal gene clusters (Badger and Bek, 2008). It is interesting to note that the photosynthesizers dominating our planet's landmass, the higher plants, possess only a small slice of Rubisco's molecular diversity, all encoding a highly conserved Form IB enzyme derived from the ancestral cyanobacterial endosymbiont.

Three distinct classes of Rubisco activase (green-, red-, and CbbQO-type) have now been identified (Salvucci et al., 1985; Mueller-Cajar et al., 2011; Tsai et al., 2015), permitting us to start dissecting the molecular underpinnings of how different organisms dealt with the outlined problem of blocked Rubisco active sites. The activases were recruited from highly distinct volumes of sequence space in the AAA+ protein universe (Ammelburg et al., 2006), and their AAA modules display less than 25% sequence identity between the groups. This vast and diverse group of molecular motors was clearly well suited for the task of active site rearrangement, as their unifying functional characteristic relates to conformationally remodeling macromolecular substrates using the energy of ATP hydrolysis (Hanson and Whiteheart, 2005; Sysoeva, 2016). The identified activases are not closely related to other well characterized extant molecular chaperones, which currently precludes the formulation of detailed hypotheses regarding their historical evolutionary trajectory.

Green-type Rcas represent the first discovered (Salvucci et al., 1985) and due to their presence in all higher plants, most extensively studied activase system (Portis, 2003; Carmo-Silva et al., 2015). They are evolutionarily derived from cyanobacteria, where homologs are found associated with carboxysomal green-type Form IB Rubisco (Li et al., 1993). Importantly, an experimental verification of the cyanobacterial activase's biochemical function is still elusive (Bracher et al., 2017). The distribution is not universal, but is associated with strains belonging to clade A and B1 according to the classification by Kerfeld and colleagues (Shih et al., 2013; Zarzycki et al., 2013). These are thought to form the sister group to the primary endosymbiont (Ochoa de Alda et al., 2014), which would indicate that Rca was transferred together with Form IB Rubisco during the primary endosymbiotic event about 1.5 billion years ago (Yoon et al., 2004).

On a structural level green-type Rcas show similarity to p97/CDC48 (Hasse et al., 2015) and classification of the C-terminal subdomain revealed a relationship to the D2 AAA+ module of N-ethylmaleimide-sensitive factor (NSF) (Ammelburg et al., 2006). Both of these belong to the classical clade of AAA proteins (Iyer et al., 2004). It is thus reasonable to conclude that specialization toward activase activity occurred using a general molecular chaperone in this clade in an ancient cyanobacterium as a starting point.

The gene encoding red-type Rca (also known as CbbX), is always found in an operon with the red-type (Form IC) Rubisco encoding genes in mixotrophic proteobacteria (Gibson and Tabita, 1997; Badger and Bek, 2008). It is also encountered in the chloroplast genomes of the red lineage (Oudot-Le Secq et al., 2007). A proposed explanation for this distribution involved horizontal gene transfer of the *rbcL*-*rbcS*-*cbbX* gene cluster from a proteobacterium to an ancestor of the primary endosymbiont (Delwiche and Palmer, 1996; Nisbet et al., 2004). Alternatively horizontal gene transfer occurred subsequent to the endosymbiotic event in the ancestor of the red algae, which subsequently lost the green Form IB Rubisco genes (Maier et al., 2000; Rice and Palmer, 2006). Where sequence data exists, it appears eukaryotes possessing red-type Rubisco always encode an additional CbbX isoform in the nuclear or nucleomorph genome (Hovde et al., 2015), and this is thought to be a consequence of gene duplication and migration of one copy to the nuclear genome in an early rhodophyte (Fujita et al., 2008). In the red algae *Cyanidioschyzon merolae*, the functional red-type Rca has been shown to be a 1:1 hetero-oligomer of the plastid and the nuclear encoded isoform (Loganathan et al., 2016), and we expect this scenario to hold true for red lineage phytoplankton in general.

The closest structural neighbors of red-type Rca, as determined by a DALI search are the helicase RuvB and protease-associated motors such as HslU and ClpX (Hasse et al., 2015). HslU and ClpX are powerful unfoldases that generally thread substrate proteins marked for degradation through their axial pore of the hexamer into a proteolytic chamber (Sauer and Baker, 2011). However, recently more gentle conformational rearrangements have been documented for the mitochondrial ClpX. In this case ClpX acts on an enzyme involved in heme biosynthesis and catalyzes the insertion of a cofactor (Kardon et al., 2015). Hence it is conceivable that subtle “pulling” on enzymes to bring about conformational transitions that favor inhibitor release or co-factor insertion is not an unusual scenario (Olivares et al., 2016). It is therefore a reasonable hypothesis that red-type Rca evolved in proteobacteria from a general molecular chaperone using the axial pore threading mechanism that was either involved in correcting protein conformations or protein complex maturation (including co-factor insertion).

The genes encoding the CbbQO-type activase system (Hayashi et al., 1997, 1999; Sutter et al., 2015; Tsai et al., 2015) are broadly distributed among proteobacteria, but associate strongly with chemolithoautotrophs that use sulfur oxidation as energy source (Badger and Bek, 2008). CbbQ belongs to the large, but relatively poorly characterized MoxR group of AAA+ proteins, which is often found encoded in operons together with a second protein containing a von Willebrand Factor A (VWA) domain (Snider and Houry, 2006; Wong and Houry, 2012).

Different isoforms of the AAA+ protein CbbQ and the VWA-domain containing CbbO assemble as hetero-oligomeric complexes in a Q_6_O_1_ stoichiometry (Sutter et al., 2015; Tsai et al., 2015). Two complexes encoded by *Acidithiobacillus ferrooxidans* activate phylogenetically remote Rubiscos (Q1O1 activates Form IA^Q^ and Q2O2 activates Form II) that are encoded by the same genome (Tsai et al., 2015). In addition there is a third *cbbQ-cbbO* gene pair (termed Q3O3 in Figure 2) associated with a carboxysomal gene cluster, which contains genes encoding a Form IA^C^ Rubisco (Heinhorst et al., 2002). The activase function of Q3O3, which is homologous to a complex recently purified and characterized for ATPase activity, has not yet been confirmed (Sutter et al., 2015). This work also pointed out that the presence of multiple Rubisco operons encoding different CbbQ and CbbO isoforms in the same organism is common. It is thus possible that the ancestor of the CbbQO complex became specialized for one Rubisco form, and then switched substrate following a gene duplication. Alternatively the ancestral CbbQO was a generalist Rca and already functional at remodeling both types of Rubisco. The feasibility to reconstruct ancestral proteins offers a tantalizing opportunity to illuminate these details experimentally (Shih et al., 2016).

Gene pairs highly homologous to CbbQ and CbbO that are not associated with Rubisco genes also exist in proteobacteria (Snider and Houry, 2006; Sutter et al., 2015). The genes encoding the AAA+ protein NirQ and VWA domain protein NorD, are associated with denitrification gene clusters. In the absence of either NirQ or NorD, nitric oxide reductase is produced in non-functional form, implicating NirQ-NorD in enzyme maturation or assembly (Jungst and Zumft, 1992; de Boer et al., 1996). The best biochemically characterized MoxR AAA+ ATPase chaperone system is RavA-ViaA, where RavA is the AAA+ motor, and ViaA is an interacting VWA-domain containing protein (Snider et al., 2006; Wong et al., 2017). Intriguingly one of a number of described function of RavA involves a reduction of the affinity of the allosteric inhibitor ppGpp to the enzyme lysine decarboxylase (albeit in a ViaA independent manner) (El Bakkouri et al., 2010; Kanjee et al., 2011). Therefore, it is likely that in this family many chaperones with functions related to the modulation of enzyme activity remain to be discovered. The CbbQO Rubisco activation system was likely derived from such an origin.

## The architecture of inhibited rubisco active sites

It is established that contemporary Rubisco enzymes all share a common ancestor (Tabita et al., 2007), and although there is significant diversity in quaternary structure, tertiary structure is essentially conserved (Andersson, 2008; Andersson and Backlund, 2008). The implication is thus that the different Rca motors will encounter a highly similar substrate, irrespective of its origin. It is therefore reasonable to expect that Rca mechanisms will display similarities. Consequently motor-substrate specificity should be exchangeable by targeted mutagenesis once the mechanisms are understood in sufficient detail.

**Figure 3:**
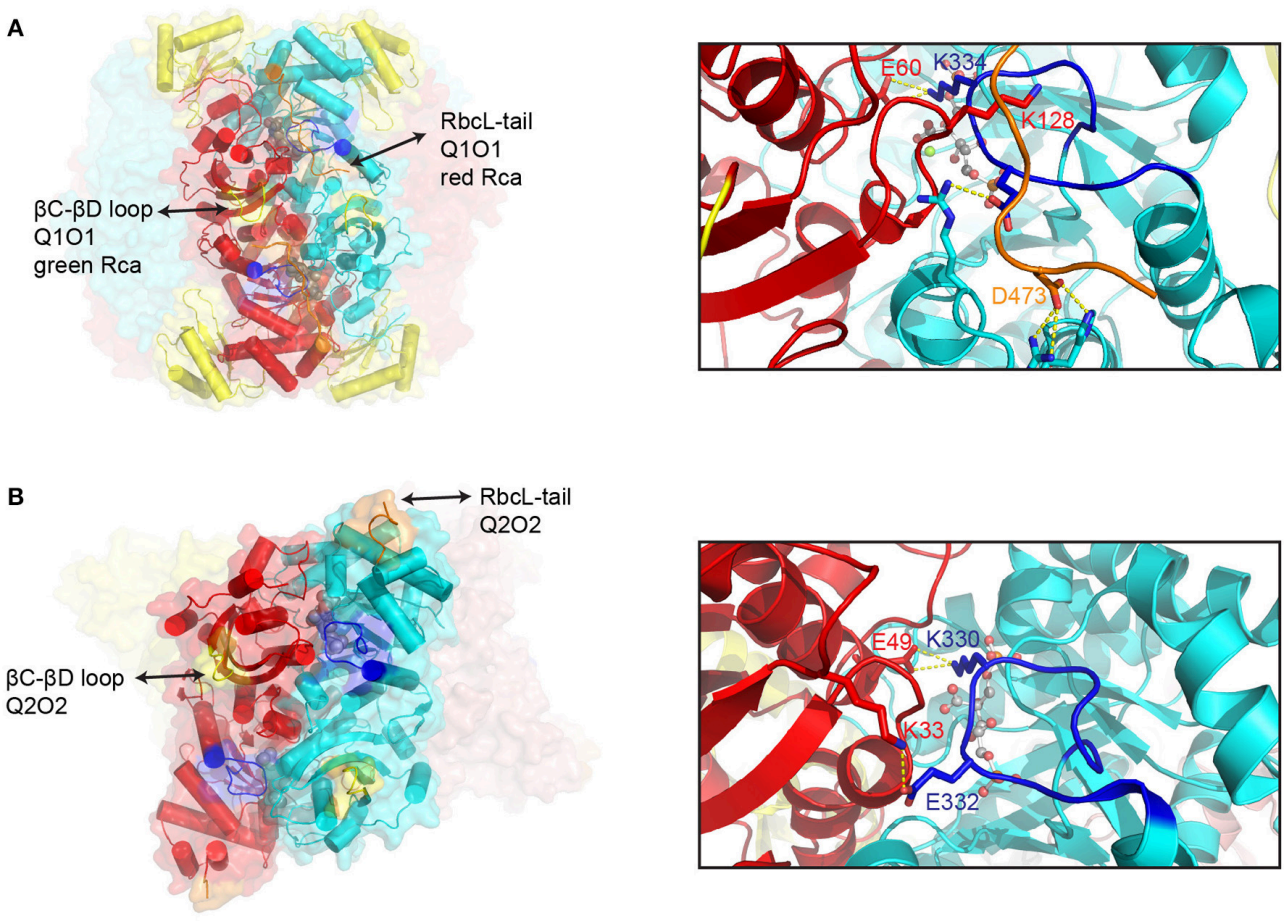
**Structural features of inhibited Rubisco complexes**. A comparison of structural elements involved in the Rca-mediated activation of Form I **(A)** and Form II **(B)** Rubisco. Left panels: Surface representation of CABP-bound spinach (pdb:8ruc) and *R. palustris* Rubisco (pdb:4lf1). One large subunit dimer pair (in red and cyan) is shown with helices represented by cylinders. Key segments are colored as follows: βC-βD loop, yellow; Loop 6, blue; C-terminal strand, orange. Right panels: Close-up of the active site highlighting differences in Loop 6 (in blue) closure between Form I and Form II Rubisco. Key residues and interactions are highlighted. Bound CABP is shown in ball and stick representation. The following indicated residues are conserved and functionally equivalent (Form I/Form II): E60/E49; K334/K330).

Representative examples of Form I and Form II inhibited Rubisco complexes that function as Rca substrates are shown in Figure 3. The active site is located at the C-terminal face of the beta strands forming the αβ barrel. Residues contributing to the active site are mostly found in the loops connecting the beta strands of the barrel to the downstream helices, but a few are donated by the N-terminal domain of the opposing subunit. Once the substrate RuBP has bound, loop 6 of the beta barrel folds over the active site to form the closed state (Karkehabadi et al., 2007). Loop 6 contributes a critical lysine residue (Form I- K334, Form II- K330), which is thought to position the CO_2_ molecule for carboxylation. In Form I enzymes, closure of the active site is accompanied by the C-terminal strand of the large subunit folding over loop 6, with Asp-473 believed to act as a latch residue (Duff et al., 2000; Satagopan and Spreitzer, 2004). The thus secured C-terminus is envisaged to be under tension to push down on Loop 6 via Lys-128 (Bainbridge et al., 1998), providing rigidity to the carboxylation ready active site (Duff et al., 2000). In stark contrast to the C-terminal locking mechanism in Form I Rubisco, inspection of the closed form of the carboxy-arabinitol 1,5 bisphosphate (CABP) bound Form II hexamer from *Rhodopseudomonas palustris* reveals that the C-terminus does not fold over and lock down Loop 6, but is instead positioned at the apex of the complex (Satagopan et al., 2014) (Figure 3B). As a consequence Loop 6 is surface exposed in these structures (Satagopan et al., 2014; Varaljay et al., 2016). Instead of being held in place by the C-terminus, the structure reveals a salt-bridge between Glu-332 (*R. palustris* RbcL labeling) and Lys-33 on the opposite subunit. These residues are conserved in many Form II enzymes, and the interaction may thus be part of an alternative Loop 6 locking mechanism. Another important feature of active site closure concerns a 2° rotation of the N-terminal domain, resulting in a reduced distance between the phosphate binding sites of the active site (Taylor and Andersson, 1996; Duff et al., 2000).

Based on these observations, the conformational changes to bring about an opening of the active site catalyzed by the Rca motors could either involve manipulation of the C-terminal domain, for instance by disruption of the latched C-terminus in Form I enzymes, or Rca-induced movement of the N-terminal domain. In fact both strategies appear to be utilized.

## Oligomeric state and regulation of the activases

The three classes of Rca identified so far all belong to distantly related branches of the AAA+ protein superfamily and possess a single AAA+ domain. Experimentally determined atomic models of the AAA+ module of all activase classes are now available, and all exhibit the expected architecture of this protein family (Henderson et al., 2011; Mueller-Cajar et al., 2011; Stotz et al., 2011; Hasse et al., 2015; Sutter et al., 2015). A Rossmann fold forms the nucleotide binding domain, which is followed by a small α-helical subdomain (Erzberger and Berger, 2006, Figure 4A). AAA+ proteins commonly form hexameric rings, and this is certainly the functional form of both the red-type (Mueller-Cajar et al., 2011; Loganathan et al., 2016) and the CbbQO-type Rcas (Sutter et al., 2015; Tsai et al., 2015) as verified by negative-stain electron microscopy.

**Figure 4:**
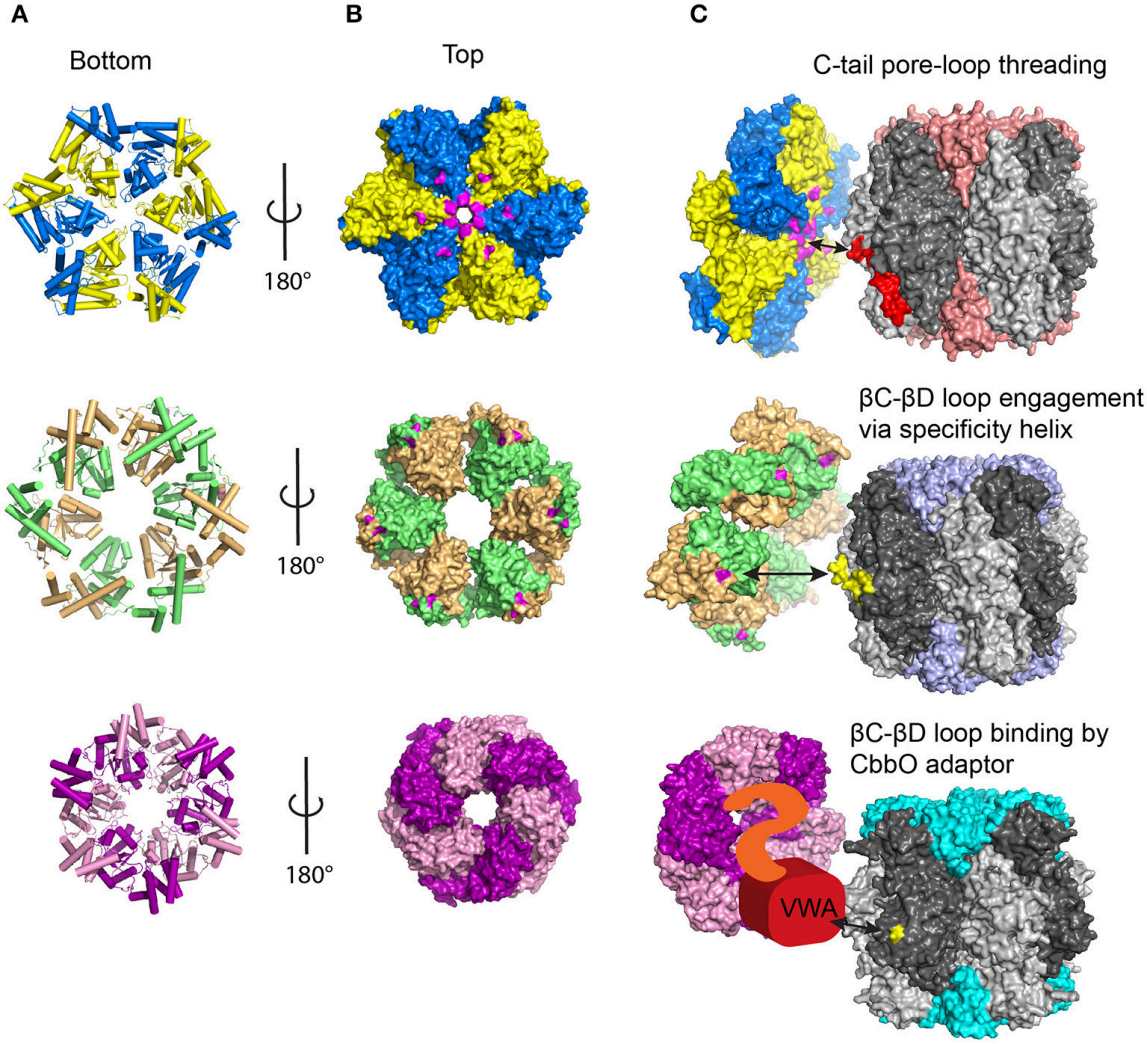
**Current models of Rubisco activase function. (A)** Bottom view of the different Rca hexameric models showing helices in cylinder view. Adjacent subunits are colored differently **(B)** Top view of the Rca models in surface representation. Residues known to be involved in protein-protein interactions with Rubisco are colored in magenta for red and green-type Rca. **(C)** Current mechanistic models for the different Rca systems. See text for details. Known Rca interacting segments on Rubisco are shown in red (RbcL C-tail) and yellow (interacting βC-βD loop residues). Red-type Rca /Rubisco, PDB:3ZUH/1BXN; Green-type Rca/Rubisco PDB:3ZW6/8RUC; CbbQ/Form IA Rubisco, PDB:5C3C/1SVD.

It is interesting to note that the proteobacterial red-type Rca forms an ATPase inactive fibril in the presence of Mg-ATP. Binding of Rubisco's substrate RuBP to a pocket located in the α-helical subdomain triggers an oligomeric transition to the ATPase and activase functional hexamer (Mueller-Cajar et al., 2011). In contrast the enzyme from the red algae *Cyanidioschyzon merolae* presents as a constitutive hexamer composed of alternately arranged nuclear and plastid-encoded isoforms (Loganathan et al., 2016). However, the RuBP-binding pocket is conserved in both isoforms and ATPase activity is stimulated by the addition of RuBP. Thus, in both prokaryotes and eukaryotes enzymatic activity of red-type Rca is allosterically regulated by the substrate of the remodeller's target. Nevertheless, mutational studies indicated that the two red-type Rca isoforms in red algae are functionally non-equivalent. For instance eliminating ATPase function of the plastid-encoded isoform by mutating the conserved Walker B glutamate to glutamine counterintuitively enhanced ATP hydrolysis of the hetero-oligomeric complex and resulted only in slight impairment of activase function. In contrast the equivalent substitution in the nuclear encoded isoform eliminated both Rca and ATPase function (Table 1). It remains to be seen whether these specializations have resulted in genuine enhancements in activase function or whether they are manifestations of molecular ratchet- type evolutionary trajectories (Gray et al., 2010; Finnigan et al., 2012).

**Table 1:** **Overview of selected key Rca and Rubisco mutants providing insights into the activation mechanism and listed in the order referred to in the text**.

**Protein**	**Mutation**	**Result/Interpretation**	**Reference**
*C. merolae* CmP (red-type, red algal Rca)	Walker B-E138Q	Functional CmNP hetero-oligomer/CmP has mostly structural role	Loganathan et al., 2016
*C. merolae* CmN (red-type, red algal Rca)	Walker B-E138Q	Inactive CmNP heterooligomer/CmN ATPase critical	Loganathan et al., 2016
*C. merolae* CmN/CmP and *R. sphaeroides* RsRca	Pore loop 1 tyrosine- Y114A	ATPase functional, Rca inactive/pore-loop treading mechanism	Mueller-Cajar et al., 2011; Loganathan et al., 2016
*R. sphaeroides* RbcL (Form IC Rubisco)	C-terminal deletions (Δ2, Δ4)	Rubisco functional, but cannot be activated by Rca/ C-terminal threading mechanism	Mueller-Cajar et al., 2011; Loganathan et al., 2016
*Nicotiana tabacum* RbcL	C-terminal extension by His_6_-tag	Rubisco functional and can be activated by Rca/likely no C-terminal threading mechanism	Scales et al., 2014
*N. tabacum* Rca	Specificity helix, D316K, L319V double mutant	Gains ability to activate Spinach Rubisco/βC-βD loop engagement mechanism	Li et al., 2005
*N. tabacum* Rca	Pore loop 1/Pore loop 2, A144V, Y188A	ATPase functional, Rca inactive/pore loop threading mechanism	Stotz et al., 2011
*Chlamydomonas reinhardtii* RbcL	βC-βD loop, D94K, P89A/R	Mutants gain ability to be activated by tobacco Rca/βC-βD loop engagement mechanism	Larson et al., 1997; Ott et al., 2000
*N.tabacum* Rca	N-terminal deletions (ΔN51, ΔN58)	ATPase functional, Rca inactive/N-terminal domain required for engagement	Esau et al., 1996; van de Loo and Salvucci, 1996; Stotz et al., 2011
*A. ferrooxidans* RbcL (Form I and Form II)	Multiple C-terminal modifications to probe H/KR motif function	Rubisco functional but activation by Rca impaired or eliminated/C-terminal engagement mechanism	Tsai et al., 2015
*A. ferrooxidans* CbbQ2	Multiple pore loop mutations	Rca function not perturbed/C-terminal threading mechanism does not apply	Tsai et al., 2015
*A. ferrooxidans* CbbO2	MIDAS motif- D573A, S575A, S577A, T656A, D684A	ATPase functional, Rca function eliminated or strongly perturbed/implicates MIDAS in Rca-Rubisco interaction	Tsai et al., 2015
*A. ferrooxidans* RbcL (Form I and Form II)	βC-βD loop homologous acidic residue- D82P (Form I), E75A (Form II)	Rubisco functional but activation by Rca impaired (Form II) or eliminated (Form I)/ βC-βD loop engagement mechanism	Tsai et al., 2015
*A. ferrooxidans* CbbO2	C-terminal deletion (residues 445–759)	Rca non-functional, Complex formation intact/residues 1–444 interact with CbbQ_6_ hexamer	Tsai et al., 2015

The *in vitro* oligomeric state of the green-type Rcas is highly polydisperse, possibly ranging from monomeric (Keown et al., 2013) to very large assemblies (Barta et al., 2010; Chakraborty et al., 2012; Kuriata et al., 2014). However, the existence of functional, stable hexamers (Blayney et al., 2011; Stotz et al., 2011; Keown and Pearce, 2014) suggest that this is also the functional species. It is possible that the oligomeric forms may be transitional to permit efficient movement of the activases through the extremely crowded chloroplast stroma (Harris and Koniger, 1997), permitting this less abundant helper protein to shuttle between inactive Rubisco active sites as required. Hexameric assemblies would then occur transiently to form the functional assembly at the inhibited substrate Rubisco. Consistent with this notion, green-type activases rapidly exchange subunits *in vitro* (Salvucci and Klein, 1994; van de Loo and Salvucci, 1998; Stotz et al., 2011). Regulation of the green-type Rca in higher plants is complex (Carmo-Silva and Salvucci, 2013; Hazra et al., 2015), with a number of mostly energy-related signals integrating. These include redox modulation by thioredoxin and inhibition by ADP (reviewed by Carmo-Silva et al., 2015 and Portis, 2003) and most recently reversible phosphorylation (Boex-Fontvieille et al., 2014; Kim et al., 2016).

CbbQO is unique among activases, in that the AAA+ hexamer CbbQ associates with a single adaptor protein CbbO, which is essential for activase function. The CbbQ_6_O_1_ complexes are monodisperse and do not disassemble as assessed by gel filtration chromatography (Tsai et al., 2015). Finally, both CbbQO and red-type Rubisco activases exhibit a strong stimulation of their ATPase activity when assayed in the presence of inhibited Rubisco complexes (Mueller-Cajar et al., 2011; Tsai et al., 2015; Loganathan et al., 2016). This type of regulation is not observed in the green-type Rcas (Robinson and Portis, 1989; Hazra et al., 2015).

## Mechanistic insights into rubisco remodeling

AAA+ proteins generally function by translating conformational changes brought about by ATP hydrolysis to a macromolecular substrate, and this principle applies to Rcas and Rubisco. The best described mechanisms so far involve the translocation of the substrate through the axial pore of the hexameric AAA+ ring. This involves a conserved pore loop 1 tyrosine in many well-studied systems, including ClpX (Siddiqui et al., 2004), ClpB/Hsp104 (Weibezahn et al., 2004) and the AAA+ unfoldase of the proteasome (Beckwith et al., 2013). In Table 1 I summarize biochemical evidence for the mechanistic models described in this section. The outlined threading mechanism appears to be utilized by red-type Rca in both photosynthetic bacteria and red algae. In this model, the activase transiently threads the C-terminus of the Rubisco large subunit into the pore (Figure 4C). Red-type Rubiscos all appear to possess a C-terminal extension of 11–12 residues following the critical latch residue Asp-473, which locks the C-terminus to its large subunit. Thus, by pulling on this peptide, the interaction of Asp-473 with its own subunit can be disrupted, releasing the lock and allowing loop-6 to retract, followed by release of the bound inhibitor. Substitutions with alanine of the conserved pore loop 1 tyrosine in both the bacterial and algal red-type Rca, as well as two and four amino-acid deletions of the RbcL C-terminus abolish activase function (Mueller-Cajar et al., 2011; Loganathan et al., 2016).

Interestingly this model, at least relating to transient threading of the Rubisco large subunit C-tail, is unlikely to apply to either of the other two Rca classes. In contrast to the red-type Rubiscos, the C-termini of green-type Rubiscos are of variable length, but often only have 2–4 residues following the latch residue (Satagopan and Spreitzer, 2004). Green-type Rca is thus unlikely to engage this short and variable motif. It was also found that an extension of the tobacco large subunit by six histidine residues did not affect Rca function (Scales et al., 2014). In addition the central pore of the green-type Rca hexamer has a larger diameter than that of red-type Rca, which lead to the hypothesis that a larger secondary structural element, such as a loop, could be threaded instead (Stotz et al., 2011). Consistent with the general theme of a poreloop threading mechanism mutational analysis of pore loop 1 and 2 resulted in the discovery of variants that maintained ATPase function but no longer activated Rubisco (Stotz et al., 2011). Notably, the AAA+ chaperone ClpB has been demonstrated to be capable of threading a looped segment (Haslberger et al., 2008), and the threading mechanism is therefore not limited to free N or C-termini.

The surface exposed βC-βD loop of the large subunit N-terminal domain has long been implicated in the interaction with green-type Rca (Figure 3A). Residues 89 and 94 (spinach numbering) in this loop are known to interact with residues 316 and 319 (tobacco Rca numbering) of the activase (Larson et al., 1997; Ott et al., 2000; Li et al., 2005), which are located on a helical insertion in the small subdomain of the AAA+ module (Stotz et al., 2011; Hasse et al., 2015). This interaction involves the same (top) face of the disc-shaped hexamer that is involved in red-type Rca function (Wachter et al., 2013, Figure 4B). In addition an N-terminal domain of ~70 amino acids is also involved in the Rubisco-Rca interaction (Esau et al., 1996; van de Loo and Salvucci, 1996; Stotz et al., 2011), however it is not resolved in current crystal structures. It is conceivable that following initial engagement by activase involving the mentioned structural elements (Figure 4C), a pulling force to the βC-βD loop could be brought about by Rca pore loop threading. Rigid body movement of the attached beta sheet would then result in the rotation of the N-terminal domain seen when comparing the closed and open form of the enzyme (Duff et al., 2000).

Mutational analysis of both CbbQO and the two different classes of substrate Rubisco revealed the basis of a common mechanism for CbbQO-type Rcas. More fascinatingly, the results revealed commonalities to both red- and green-type Rca function. It was noted that in spite of low (~30%) primary sequence identity of the Form I and Form II Rubisco large subunits, the C-termini of those enzymes encoded in *cbbQ*-*cbbO* containing gene clusters displayed a common C-terminal sequence motif (H/KR). Mutagenesis of this motif strongly impaired the ability of the target Rubiscos to be activated by their activases, drawing a strong mechanistic parallel to the pore-loop threading red-type Rcas (Tsai et al., 2015). However, experiments attempting to perturb the poorly conserved pore-loop region of CbbQ did not result in non-functional Rca, and I currently favor a model where the large subunit C-terminus is bound (and consequently immobilized) by the activase, rather than threaded. Here I am also considering the fact that in the Form II substrate the C-terminus does not occupy the same locked latch position as in the Form I complex (Figure 3, Satagopan et al., 2014), and thus exerting a pulling force on this motif would not have the same effect.

As is commonly observed for the MoxR class of AAA+ proteins, the CbbO adaptor encoded downstream of the *cbbQ* gene possesses a von Willebrand factor A (VWA) domain at its C-terminus (Whittaker and Hynes, 2002). This well-described protein-protein interaction module generally uses four residues that are part of a motif known as metal ion dependent adhesion site (MIDAS) to bind a divalent cation. Mutating conserved MIDAS residues mostly abolished CbbQO activase function (Tsai et al., 2015). A fifth ligand to the divalent cation is generally donated by an acidic residue of the interacting protein (Xiong et al., 2002; Santelli et al., 2004). It was discovered that mutating a conserved acidic residue in the previously mentioned surface exposed βC-βD loop of the Rubisco large subunit N-terminal domain to alanine abolished (Form I Rubisco) or greatly reduced (Form II) the ability of Rubisco to become activated by CbbQO (Tsai et al., 2015). Fascinatingly this residue is at the same position as the green-type Rca interacting residue 89 in higher plants Rubisco. We therefore predict that the ATP-hydrolysis powered conformational change brought about by CbbQO and green-type Rcas will emerge to be similar in nature (Figure 4C). The precise interaction between a CbbQ hexamer and the CbbO adaptor has not been resolved so far, but involves residues 1–444 of CbbO (Tsai et al., 2015). It is possible that the conformational changes of the hexamer generated by ATP hydrolysis are transmitted to the VWA domain via the CbbO N-terminal region (Figure 4C).

Disruption of the closed conformation of the Rubisco holoenzyme by Rca of all three classes will lead to release of the inhibitory sugar phosphate. The active site is thus reset either for cofactor binding, or acceptance of the substrate RuBP (if the inhibitor removed was already bound to ECM holoenzyme, Figure 1C).

## The role of the activases in a synthetic biology of CO_2_ fixation

A strong impetus regarding research into the detailed mechanisms underlying Rubisco repair in autotrophic organisms is provided by the realization that relatively poor Rubisco performance contributes to the low photosynthetic efficiency of plants, and enhancing its activity is predicted to significantly improve the yield of crops (Long et al., 2015). Given the tight coupling of carboxylase function to maintenance of its activation state by the described highly diverse Rca proteins, any modifications of Rubisco will need to keep in mind compatibilities and other properties of Rca.

## Rubisco and Rca transplantation

A number of strategies regarding the enhancement of C3 photosynthesis rely on the concept of transplanting a Rubisco enzyme of choice into a target crop (Andrews and Whitney, 2003; Zhu et al., 2004). Such experiments need to ensure the presence of a suitable Rca, and technically this is not a difficult problem. Rca in higher plants is encoded by the nuclear genome, and thus *Agrobacterium tumefaciens* based transformation methods can successfully deliver a target *Rca* gene (Kurek et al., 2007; Kumar et al., 2009; Fukayama et al., 2012). Deletion or silencing of the endogenous *Rca* genes may be advantageous if hetero-oligomerization is likely to occur (for instance if a green-type Rca is to be transplanted). In particular the rapid development of CRISPR-Cas9 technology will facilitate this process further (Belhaj et al., 2015). However, the relative ease of Rca engineering does not extend to Rubisco. Since in higher plants the rubisco large subunit is encoded by the chloroplast (as opposed to the nuclear) genome, this achievement requires the replacement of the endogenous *rbcL* genes in multiple plastid genome copies. Following significant technical progress in the past decades it is now possible to routinely perform this experiment in tobacco plants using biolistic transformation. Here a particular boon has been the development of a marker-free tobacco-rubrum “master” line (Whitney and Sharwood, 2008), which has its endogenous hexadecameric Form IB Rubisco replaced by a bacterial dimeric Form II enzyme. Due to this Rubisco's low CO_2_/O_2_ specificity, it only permits plant growth at elevated levels of CO_2_ (Whitney and Andrews, 2001) and thus facilitates the isolation of transformants expressing more catalytically adept heterologous Form I enzymes. Key examples of successful rubisco transplantation experiments include various higher plant enzymes (Sharwood et al., 2008; Whitney et al., 2011b, 2015), a cyanobacterial Form I enzyme (Lin et al., 2014b) and an archaeal Form III enzyme from *Methanococcus burtonii* (Wilson et al., 2016). It is therefore technically feasible to produce functional heterologous Rubisco in tobacco plants, although expansion of the technology to other species has so far met with modest success and most crops cannot currently be modified in this manner (Bock, 2015). Current efforts in this area of research are aiming to identify better suited higher-plant Rubiscos (Orr et al., 2016; Sharwood et al., 2016a), or introducing single residue changes into the large subunit that result in desired catalytic switches (Whitney et al., 2011b). Here activase requirements should be easy to satisfy due to the wide level of compatibility between plant Rubiscos and green-type Rcas (Wang et al., 1992). Still a relative paucity of Rubisco-Rca compatibility data may require careful biochemical characterization on a case to case basis.

Although production of heterologous Rubisco in higher plants is currently feasible, a key limitation concerns our incomplete understanding of the enzyme's folding and assembly machinery, which results in either low Rubisco content, or a complete failure in functional Rubisco expression. Regarding the production of heterologous plant Rubisco, rapid progress is being made, for instance co-expression of the Rubisco assembly chaperone Raf1 (Feiz et al., 2012; Hauser et al., 2015a) permitted a doubling of correctly assembled Arabidopsis Rubisco large subunits in tobacco chloroplasts (Whitney et al., 2015).

Among the most tempting targets for transplantation are the red-type Form ID Rubiscos from red algae, some of which have evolved CO_2_/O_2_ specificity values that are twice as high than those found in the land plant Form 1B enzymes (Read and Tabita, 1994; Uemura et al., 1997). For instance functional production of the Rubisco from the red algae *Griffithsia monilis* (Whitney et al., 2001) in higher plant chloroplasts is predicted to result in a 27% increase in daily canopy carbon gain (Zhu et al., 2004). However, early experiments to produce these proteins in tobacco led to complete insolubility of the gene products (Whitney et al., 2001), consistent with an incompatibility of the folding and/or assembly chaperone machinery. Interestingly this apparent dependency on sophisticated chaperone machinery does not extend to the related bacterial Form IC red-type Rubiscos. The enzyme from *Rhodobacter sphaeroides* has no requirements for assembly chaperones, merely requiring the GroEL-ES chaperonin for productive folding of the large subunit in a reconstituted system (Joshi et al., 2015). Meeting the biogenesis requirements of Form ID Rubisco may thus be less complicated than that of the higher plant Form IB enzymes, which appear to require a plethora of assembly factors including Raf1, Raf2 and possibly RbcX (Liu et al., 2010; Feiz et al., 2014; Bracher et al., 2017). Once Form ID Rubisco transplantation has been achieved it will need to be supplemented with a red-type Rubisco activase. Based on the work with purified *C. merolae* proteins it is likely that the cognate algal Rca, a hetero-oligomer of nuclear and plastid encoded subunits, will be optimal for this purpose. However, the simpler homo-oligomeric bacterial red-type Rcas also presents with some activity toward the algal enzyme and thus may be sufficient (Loganathan et al., 2016).

A challenging goal that is currently being pursued by a number of groups involves the transplantation of the prokaryotic carboxysomal CO_2_-concentrating mechanism into the higher plant chloroplast (Price et al., 2008; Lin et al., 2014a,b). A combination of a high velocity Rubisco operating at very high CO_2_ concentrations achieved by carboxysomal Rubisco compartmentalization and active inorganic carbon transport should permit high carbon dioxide assimilation in the absence of photorespiration (Zarzycki et al., 2013). When considering this strategy it is important to realize that a subset of carboxysomal gene clusters include homologs of all three classes of Rca (Zarzycki et al., 2013; Sutter et al., 2015). Activase activity has not yet been demonstrated for any of the carboxysomally associated Rcas biochemically, and an inability to detect this function biochemically was reported in two cases (Li et al., 1999; Sutter et al., 2015). However, in my opinion the association of these Rca homologs with carboxysomal gene clusters is indicative that the associated Rubiscos have not escaped from the activase dependency. Progress here will likely require the use of Rubisco inhibitors other than RuBP, which binds only weakly to carboxysomal Rubiscos (Andrews and Abel, 1981; Pearce, 2006), as well as assay conditions that mimic the crowded carboxysomal interior. In order for Rca associated carboxysomes to function optimally, the relevant activase will likely also need to be supplied (Long et al., 2016).

It is intriguing that significant numbers of carboxysome-containing organisms do not appear to encode Rca proteins (Zarzycki et al., 2013), suggesting either a true activase independence or the existence of unidentified activase classes. Another enticing possibility would involve members of the general chaperone machinery functioning as activases, in a scenario resembling the situation prior to the evolutionary recruitment of specialized Rcas.

## Overcoming the thermolability of Rca

For a long time it has been realized that plant photosynthesis is highly sensitive to temperature stress (Berry and Bjorkman, 1980), and that the reduction of this process was correlated with a loss in Rubisco activation state (Weis, 1981; Kobza and Edwards, 1987). The discovery that Rca is highly thermolabile, and undergoes heat denaturation at physiologically relevant temperatures provided a mechanistic basis to this observation (Feller et al., 1998; Crafts-Brandner and Salvucci, 2000; Salvucci and Crafts-Brandner, 2004a). This realization was followed by the critical demonstration that expression of more thermostable Rca proteins in Arabidopsis led to enhanced growth and biomass accumulation at moderately elevated growth temperatures (Kurek et al., 2007; Kumar et al., 2009). It is therefore imperative that these promising studies are followed by rigorous analyses of crop plants expressing more thermostable Rca proteins and such experiments have been reported to be taking place (Carmo-Silva et al., 2015). It will be most important to carefully analyse such plants for deleterious phenotypes at high temperatures, since Rca thermolability has been proposed to be regulatory (Sharkey, 2005). It may thus act as a thermal fuse to bring about Rubisco deactivation under stressful high temperature conditions.

In addressing these issues clearly opportunities exist in taking advantage of more thermostable Rca proteins that exist among natural variation (Salvucci and Crafts-Brandner, 2004b; Lawson et al., 2012; Scafaro et al., 2016). It is also worth pointing out that it may not be necessary to restrict oneself to green-type Rca. The characterized red-type Rca from the thermophilic rhodophyte *C. merolae* was a functional activase at 25°C, and able to hydrolyze ATP after incubation at 60°C (Loganathan et al., 2016). Protein engineering approaches that utilize both our mechanistic insights in combination with artificial evolution experiments that utilize an expanding suite of Rubisco dependent *Escherichia coli* (RDE) systems (Mueller-Cajar and Whitney, 2008b; Durao et al., 2015; Antonovsky et al., 2016; Wilson et al., 2016) will enable incompatibilities between specific Rubiscos and activases to be overcome.

## Accelerating rubisco activation in plants

An additional opportunity to enhance Rubisco function and photosynthesis by activase engineering relates to the naturally slow activation response of Rubisco under fluctuating light conditions (Mott and Woodrow, 2000; Lawson et al., 2012). Accordingly it was shown that *Arabidopsis* plants expressing less regulated Rubisco activase isoforms were able to activate Rubisco more rapidly than wild-type plants following a dark to light transition. This property translated to increased biomass accumulation when the plants were grown under a fluctuating light regimen (Carmo-Silva and Salvucci, 2013). Rice plants overexpressing an activase from maize also displayed faster induction of photosynthesis under fluctuating light conditions (Yamori et al., 2012). These results indicate that activases that are highly functional, and thus able to rapidly convert inhibited Rubisco complexes to the ECM holoenzyme, may be able to confer enhanced photosynthetic properties to plants exposed to fluctuating light conditions that may commonly be encountered in natural environments.

While considering the possibility of qualitatively superior activases it is also worth mentioning that the thus far described members of the red-type and CbbQO type Rca clades were all able to remove the extremely tight-binding inhibitor CABP from their cognate Rubiscos (Tsai et al., 2015; Loganathan et al., 2016), whereas the green-type Rca from higher plants is unable to do so (Robinson and Portis, 1988). Although more work is required regarding the relative affinity of CABP to various enzymes, these results indicate that different clades of Rca have evolved different levels of remodeling power that can potentially be utilized to advantage in heterologous contexts.

## Outlook

It appears likely that the crops of the future will possess a photosynthetic machinery consisting of carefully selected modules that will ensure maximum yield performance in their particular environment (Zhu et al., 2010; Kromdijk et al., 2016). The properties of Rubisco and its support cast will continue to play a critical role in this endeavor (Sharwood, 2017). In order to intelligently and effectively apply modifications to the photosynthesizers of our choice, a much denser network of Rubisco and activase related data is required (Hanson, 2016). This is critical because our dependence on Rubisco as key carbon fixation catalyst will be ongoing, at least until alternative and more efficient synthetic CO_2_ fixation pathways have been successfully and fully integrated into the metabolism of photoautotrophs (Bar-Even et al., 2010; Schwander et al., 2016).

## Author contributions

The author confirms being the sole contributor of this work and approved it for publication.

## Funding

My laboratory's research on rubisco activases was funded by Nanyang Technological University (startup grant) and the Ministry of Education of Singapore (MOE2013-T2-2-089).

### Conflict of interest statement

The author declares that the research was conducted in the absence of any commercial or financial relationships that could be construed as a potential conflict of interest.
